# Hemorrhagic Shock after Neonatal Circumcision: Severe Congenital Factor XIII Deficiency

**DOI:** 10.1155/2021/5550199

**Published:** 2021-05-03

**Authors:** Erin L. Cohen, Samantha E. Millikan, Perry C. Morocco, Jill L. O. de Jong

**Affiliations:** ^1^Department of Pediatrics, Comer Children's Hospital, Pritzker School of Medicine, University of Chicago, Chicago, IL, USA; ^2^Department of Pediatrics, Section of Pediatric Hematology, Oncology, Stem Cell Transplant, Comer Children's Hospital, Pritzker School of Medicine, University of Chicago, Chicago, IL, USA

## Abstract

A Caucasian male infant born full term via normal spontaneous vaginal delivery was given vitamin K after birth, circumcised on day of life (DOL) 1, and discharged from the nursery on DOL 2. At the time of circumcision, oozing from the surgical site was noted and initially resolved with silver nitrate. Over the next two days, he presented to local emergency rooms multiple times for recurrent bleeding, eventually developing hemorrhagic shock resulting in admission to the neonatal intensive care unit. After extensive work up, he was ultimately diagnosed with severe congenital factor XIII deficiency. Congenital factor XIII deficiency is a rare bleeding disorder characterized by normal prothrombin time (PT) and activated partial thromboplastin time (aPTT) coagulation labs on routine screening, and has a high risk of complications, such as spontaneous intracranial hemorrhage. Although uncommon, when caring for a child with bleeding, physicians must have a high index of suspicion to make this diagnosis in order to initiate proper treatment and start prophylaxis given the risk of morbidity and mortality in untreated patients.

## 1. Introduction

Factor XIII deficiency is a rare bleeding disorder with an incidence of approximately one in two million births. Unlike other bleeding disorders, clots are formed normally but are unstable due to a defect in fibrin cross-linking which leads to excessive fibrinolysis. As such, patients may present with delayed bleeding after a procedure or injury, or recurrence of bleeding. Characterized by normal PT, INR, and aPTT coagulation values, the diagnosis of factor XIII deficiency is often delayed, which can result in high morbidity and mortality. Here, we present the case of an infant who experienced hemorrhagic shock after prolonged bleeding due to congenital factor XIII deficiency, which was diagnosed after multiple medical visits. A review of the scientific literature related to the clinical course, pathophysiology, and treatment options for factor XIII deficiency is also presented.

## 2. Case Presentation

The patient was a full-term infant boy born via an uncomplicated spontaneous vaginal delivery after an uncomplicated pregnancy. He was circumcised on DOL 1 after receiving vitamin K. There was oozing noted at the circumcision site after the procedure that resolved with application of silver nitrate, and he was discharged home on DOL 2. (See Table 1 for detailed chronology of the case.) Laboratory studies drawn in response to the episode of bleeding at the time of nursery discharge revealed a normal hemoglobin level (Table 2). Of note, coagulation studies (PT and aPTT) were not done prior to hospital discharge.

After arriving home, his parents took him to a local emergency room (ER) three times within 24 hours for recurrent bleeding of the circumcision site. At the initial ER visit on the night of hospital discharge, application of a Surgicel dressing controlled the bleeding. After this dressing fell off, he was brought back to the hospital a few hours later where a stitch was placed and another Surgicel dressing was applied. Later that same day, the bleeding restarted within 10 hours, leading to another ER visit for a third Surgicel dressing. Labs at this time (Tables 1 and 2) demonstrated a loss of about 2.7 g/dL of hemoglobin. There was no report of bleeding from other sites, including no bleeding from the umbilical cord stump. The bleeding appeared to be controlled, so he was discharged home with plans to follow up with his pediatrician the next day.

When he arrived to his pediatrician appointment on DOL 4, he appeared pale with weak pulses. He was transported immediately by ambulance to the nearest ER. Vital signs were notable for a heart rate of 92 bpm, respiratory rate of 20, and blood pressure of 72/59 upon arrival to the ER. He became lethargic with weak respiratory effort requiring bagging, intubation, chest compressions, emergent umbilical artery and venous catheter placement, bicarbonate, and epinephrine. Initial ER labs (Table 2) revealed severe anemia (Hgb 3.4 g/dL) and hemorrhagic shock causing severe metabolic acidosis (pH 6.415, lactate 25 mmol/L), mild thrombocytopenia (platelets 138 10^*∗*^3/uL), and a consumptive coagulopathy (PT 27.1 sec, INR 2.3, aPTT 92 sec). Fluid resuscitation was performed with 20 mL/kg of normal saline, 290 mL of packed red blood cells (approximately 90 mL/kg), and 1 unit of fresh frozen plasma (FFP). He was started on ampicillin and gentamicin due to concern for possible infection, given hydrocortisone and tranexamic acid (antifibrinolytic), and placed on a dopamine infusion for blood pressure control. With these interventions, his clinical condition stabilized, although he had continuous oozing from his circumcision site. Surgicel was again placed on the site, with a thrombin soaked gauze applied over the Surgicel.

At this point, he was transferred to a level IV neonatal intensive care unit at a tertiary care center for escalation of care. Upon arrival, he was immediately evaluated by pediatric surgery and urology and taken to the operating room where diffuse oozing was noted from the circumcision site. Hemostasis was obtained, and the patient did well postoperatively with no bleeding recurrence. Head and abdominal ultrasounds were performed and did not reveal other sites of bleeding. Over the next several postoperative days, he was extubated and his hemoglobin stabilized.

Further workup demonstrated an elevated factor VIII with normal factors II, V, VII, IX, X, XI, and XII for age (Table 3) [1]. Given that his PT, INR, and PTT also normalized without additional FFP, workup was sent to evaluate for bleeding disorders with normal PT and aPTT values, including von Willebrand disease, plasminogen activator inhibitor-1 (PAI-1) deficiency, and factor XIII deficiency. PAI-1 antigen level and von Willebrand disease panel were normal.

Ultimately, the quantitative factor XIII activity, a chromogenic assay performed by ARUP laboratories, was found to be low at 20% (age-adjusted mean and standard deviation 89 ± 24) [1], raising concern for a congenital factor XIII deficiency. Given recent FFP administration, this number was thought to be artificially elevated, so repeat testing was performed 2 months after discharge which confirmed the diagnosis with an undetectable (<5%) factor XIII activity level. Genetic testing performed by Machaon Diagnostics revealed that he was heterozygous for a missense variant in one copy of the F13A1 gene (c.782G > A, p.Arg261His in exon 6) and was heterozygous for a different missense variant in the other copy of the F13A1 gene (c.610G > A, p.Glu204Lys in exon 5), confirming a deficiency due to compound heterozygous factor XIII subunit A mutations. He is currently receiving monthly recombinant factor XIII prophylaxis and has had no further episodes of bleeding.

## 3. Discussion

### 3.1. Pathophysiology

**Figure 1:**
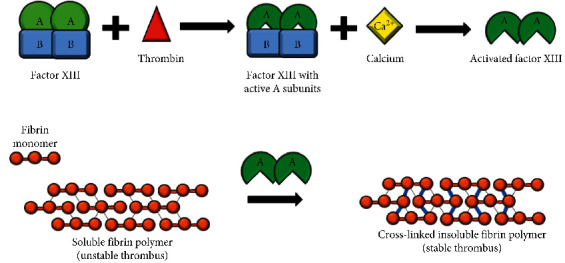
Diagram of factor XIII activation and function. (a) Factor XIII is a protransglutaminase zymogen heterotetramer composed of two catalytic A subunits and two carrier B subunits. Thrombin cleaves the A subunits to make them catalytically active, followed by calcium-dependent dissociation of the B subunit to expose the active enzymatic site on the A subunit. (b) Activated factor XIIIa is then able to crosslink the fibrin strands, by catalyzing the formation of covalent bonds between glutamine and lysine residues on the fibrin a and g chains, resulting in a stable thrombus that is more resistant to degradation and less soluble.

Factor XIII is a protransglutaminase composed of two catalytic A subunits and two carrier B subunits (Figure 1). When activated by thrombin and calcium, factor XIII plays an important role in the final stage of the coagulation pathway, with its primary function being to stabilize newly formed clots by cross-linking the fibrin strands [2]. In addition to protecting the fibrin clot against fibrinolysis, factor XIII plays an important role in wound healing and maintenance of pregnancy [2, 3]. Factor XIII deficiency is thus a disorder of secondary hemostasis, defined as involving the processes of coagulation and clot stabilization. The cutoffs for severity are controversial, with some authors defining mild deficiency when factor XIII levels are greater than 10%, moderate when 5–10%, and severe when under 5% [4], while others consider mild deficiency when over 30% and severe under 1% [5].

### 3.2. Epidemiology

Factor XIII deficiency has an incidence of approximately 1 in 2 million people, with higher prevalence in areas with higher rates of consanguineous marriage [6–8]. A 2019 report by the World Federation of Hemophilia described 1537 patients with factor XIII deficiency registered from 70 countries, with 55% males and 39% females, including 108 patients in the United States [9]. Factor XIII deficiency is categorized as a rare bleeding disorder that is most commonly congenital but can be acquired [10]. When congenital, it is inherited in an autosomal recessive pattern, distributing evenly amongst males and females, in contrast to hemophilia A and B, which are inherited in an X-linked recessive manner, thus affecting primarily males. Most cases of congenital factor XIII deficiency involve a defect in the A subunit, which can either be a quantitative type 1 deficiency with decreased protein synthesis, or qualitative type 2 deficiency with a functionally defective A subunit [11]. B subunit deficiencies are much less common, estimated to be less than 5% of cases [3]. Acquired factor XIII deficiency can be seen in many medical conditions, including leukemia, inflammatory bowel disease, cirrhosis, and thromboembolic events, and can result from increased consumption or decreased production of factor XIII or associated with antifactor XIII antibodies [11]. If due to antibody formation, systemic lupus erythematosus is a common cause, but antibodies can develop spontaneously in geriatric patients [11]. Acquired factor XIII deficiency is even rarer than the congenital form, with a 2017 review finding only 93 cases reported in the literature in adult patients [12] and only one report in a pediatric patient [13].

### 3.3. Presentation

The presentation of factor XIII deficiency can be quite variable but usually involves pathologic bleeding and often recurrence of bleeding over days. Our patient presented with prolonged bleeding from a circumcision site, with intermittent hemostasis, eventually leading to profound blood loss and hemorrhagic shock. When factor XIII deficiency is congenital, it usually presents in neonatal life, with umbilical cord bleeding being the most common presentation [14]. Other common presentations include intracranial hemorrhage, mucosal tract bleeding, spontaneous hematoma and hemarthrosis, and postsurgical bleeding [15]. Consistent with our patient's presentation of intermittent hemostasis, clots in patients with factor XIII deficiency can form normally but then undergo premature degradation after approximately 24–48 hours from poor fibrin cross-linking (Figure 1) [3]. Intracranial hemorrhage occurred in 18% of patients in one case series of 190 Iranian patients [14] and often is the initial bleed that leads to a diagnosis of factor XIII deficiency [15]. Intracranial hemorrhage is reported more frequently in factor XIII deficiency, as compared to hemophilia A and B, and is often the cause of death in patients with factor XIII deficiency [3, 11]. Another case series of 10 patients in Karachi reported prolonged bleeding and bruising in 80% of their patients, and 2 patients with prolonged bleeding from the umbilical stump [16]. Two patients had intracranial hemorrhage as the presentation [16]. Another 20-year case review from Saudi Arabia looked at 16 patients' initial presentations and found ecchymoses and recurrent hematomas in the majority of patients (71%), bleeding after circumcision as the initial presentation in 6 male patients (55%), bleeding from the umbilical stump in 7 patients (41%), poor wound healing in 3 patients (18%), and intracranial hemorrhage in 3 patients (18%) [6]. A final case series of 6 patients with factor XIII deficiency found that all presented with umbilical cord bleeding, and a significant delay in final diagnosis was noted for half of the patients, resulting in further bleeding complications [17]. Other less frequent presentations included cephalohematoma, spontaneous abortion, abruption placenta, and intraperitoneal bleeding [6]. Compared to other coagulation factors, factor XIII does not increase in pregnancy, making spontaneous abortion in the first trimester a common complication of women with severe factor XIII deficiency who did not receive prophylaxis [18].

### 3.4. Diagnosis

Any patient who presents with bleeding requires a laboratory workup that includes PT and aPTT. If these coagulation studies are normal, the differential diagnosis includes von Willebrand disease, thrombocytopenia, a platelet function disorder, PAI-1 deficiency, alpha II antiplasmin deficiency, and factor XIII deficiency [19, 20]. For patients with recurrent bleeding symptoms or intracranial hemorrhage, providers need to have a high index of suspicion for factor XIII deficiency and laboratory testing for factor XIII activity should be prioritized. Traditionally, this was done using the urea clot solubility test, which is a nonquantitative test of factor XIII activity still in use today in developing countries due to its cost-effectiveness and ease of use [21]. In developed countries, the clot solubility test is no longer recommended as it has low sensitivity for mild to moderate deficiency, including patients who are heterozygous carriers [21]. The first-line screening test for factor XIII deficiency is a quantitative factor XIII assay. Once a low factor XIII activity level is noted, the next step in diagnosis is to establish the subtype of factor XIII deficiency by measuring factor XIII-A2B2 antigen concentration in plasma, followed by factor XIII-A and factor XIII-B antigens, or through genetic sequencing of the F13A1 and F13B genes [11].

### 3.5. Treatment

In order to prevent bleeding complications, particularly spontaneous intracranial hemorrhage, patients with severe factor XIII deficiency should be started on prophylactic factor infusion [18]. The half-life of factor XIII is long at 11–14 days, and levels greater than 3% are typically sufficient to prevent bleeding [4], so only monthly prophylaxis is needed. In the event a patient with factor XIII deficiency presents with bleeding and factor XIII is not available, cryoprecipitate or FFP can be given, with cryoprecipitate having a higher concentration of factor XIII (mean 2.81 factor XIII activity per mL in cryoprecipitate versus mean 1.18 factor XIII activity per mL in FFP) [22]. As of 2021, there are currently two products approved for use in the US for factor XIII deficiency: factor XIII concentrate (human) (Corifact® by CSL Behring) and coagulation factor XIII A subunit (recombinant) (Tretten® by Novo Nordisk Inc.). Both these options have been found to be efficacious in treating factor XIII deficiency, and are overall well tolerated. An analysis of Corifact® postmarketing safety reports from 1993–2013 by CSL Behring found 75 cases of adverse events, for an estimated 1 in 15,700 standard doses [23]. Of these adverse events, 20 experienced a possible pathogen transmission, 12 a hypersensitivity reaction, 7 a possible thromboembolic event, and 5 developed a possible inhibitor [23]. For Tretten®, an international phase 3b trial exploring its safety and efficacy in pediatric patients with congenital factor XIII deficiency found that prophylaxis over 1.8–3.5 years dropped the annualized bleeding rate to zero with mostly mild adverse events [24]. Over the 16.6 patient years of the study, there were no thromboembolic events or hypersensitivity reactions observed [24]. Of note, during the study, two patients experienced a head injury and neither developed an intracranial hemorrhage [24].

## 4. Conclusion

Bleeding is an uncommon but potentially life-threatening complication after circumcision, particularly in the setting of an undiagnosed congenital bleeding disorder such as factor XIII deficiency. As coagulation labs are typically not checked prior to circumcision, parents should be counseled on the risk of bleeding complications from the procedure and the possibility (albeit rare) that the procedure itself could unmask a congenital bleeding disorder. Our patient presented on multiple occasions with refractory bleeding after circumcision and ultimately had excessive blood loss resulting in life-threatening hemorrhagic shock. Hemostasis was obtained after administration of FFP, but the diagnosis of factor XIII deficiency was delayed. Physicians must remain vigilant to diagnose congenital factor XIII deficiency in children with recurrent bleeding, particularly in the setting of the classic laboratory findings of a normal PT, INR, and aPTT.

## Figures and Tables

**Table 1:** Chronology of care.

Event	Day of life (DOL) and time
Birth	DOL 1 at 1:16 am
Vitamin K administered	DOL 1 at 8:26 am
Circumcision performed	DOL 1 at 11:52 am
Bleeding noted from circumcision	DOL 1 at 2:55 pm, 3:10 pm and 7:00 pm; unknown timing of silver nitrate application
Labs checked (Hgb 15.2 g/dL)	DOL 2 at 3:55 am
Discharged to home	DOL 2 at 4:56 pm
First visit to ER	DOL 3 at 1:11 am
Second visit to ER	DOL 3 at 4:33 am
Third visit to ER (Hgb 12.5 g/dL)	DOL 3 at 2:25 pm
Fourth visit to ER (Hgb 3.4 g/dL)	DOL 4 at 12:35 pm

**Table 2:** Laboratory values.

	Reference range	Newborn nursery (DOL 2)	3^rd^ ER visit (DOL 3)	4^th^ ER visit (DOL 4)
WBC (10^*∗*^3/uL)	6–30	15.12	10.9	8.0
Hemoglobin (g/dL)	12–20	15.2	12.5	3.4
Hematocrit (%)	36–66	46.1	36.9	12.7
Platelets (10^*∗*^3/uL)	90–475	330	312	138
RBC (10^*∗*^6/uL)	3.3–6	4.80	3.87	—
MCV (fL)	85–125	96.0	95.4	123.0
RDW (%)	11.6–14.2	16.9	15.3	17.5
nRBCs abs (10^*∗*^3/uL) [%]	0–0.1	0.22 [1.5]	—	[1]
MPV (fL)	9.0–12.6	9.4	—	—
Total bilirubin (mg/dL)	0–12	2.4	—	0.9
aPTT (seconds)	25–36	—	—	92
PT (seconds)	10.1–13.1	—	—	27.1
INR	—	—	—	2.3

ER = emergency room; DOL = day of life; WBC = white blood cell count; RBC = red blood cell count; MCV = mean corpuscular volume; RDW = red cell distribution width; nRBCs = nucleated red blood cells; MPV = mean platelet volume; aPTT = activated partial thromboplastin time; PT = prothrombin time; INR = international normalized ratio.

**Table 3:** Coagulation factor levels tested.

	Lab reference range	Newborn reference range (mean ± standard Deviation)^*∗*^	Initial results (DOL 4)	Repeat results (DOL 6)
Fibrinogen (mg/dL)	163–463	—	153	297
Factor II (%)	77–138	58 ± 14	49	57
Factor V (%)	75–137	90 ± 25	31	86
Factor VII (%)	71–147	85 ± 26	81	
Factor VIII (%)	57–152	89 ± 33	212	202
Factor IX (%)	80–147	49 ± 17	47	73
Factor X (%)	76–146	46 ± 14	42	49
Factor XI (%)	78–133	57 ± 16	—	63
Factor XII (%)	47–173	44 ± 17	—	46
Factor XIII (%)	69–143	89 ± 24	—	20

^*∗*^Source: reference [1].

## Data Availability

The data used to support the findings of this case report are included within the article.

## Abbreviations

PT: Prothrombin time
aPTT: Activated partial thromboplastin time
DOL: Day of life
ER: Emergency room
FFP: Fresh frozen plasma
INR: International normalized ratio.
